# Comparison of HIV Risk Behaviors Between Clinical Trials and Observational Cohorts in Uganda

**DOI:** 10.1007/s10461-020-02838-w

**Published:** 2020-04-10

**Authors:** Andrew Abaasa, Stephen Nash, Yunia Mayanja, Matt A. Price, Patricia E. Fast, Pontiano Kaleebu, Jim Todd

**Affiliations:** 1 MRC/UVRI & LSHTM Uganda Research Unit, Entebbe, Uganda; 2 London School of Hygiene and Tropical Medicine, London, UK; 3 International AIDS Vaccine Initiative, New York, USA; 4 Department of Epidemiology and Biostatistics, University of California at San Francisco, San Francisco, USA; 5 Pediatric Infectious Diseases, School of Medicine, Stanford University, Palo Alto, CA USA

**Keywords:** HIV, Risk behavior, Trials, Observational, Cohorts

## Abstract

Many key populations have high-risk behaviors for HIV infection making them suitable for HIV vaccine efficacy trials. However, these behaviors may change when participants enroll into a trial. We used HIV simulated vaccine efficacy trials (SiVETs) nested within observational cohorts of fisherfolks and female sex workers in Uganda to evaluate this difference. We screened observational cohort participants for enrolment into SiVETs, until 572 were enrolled. Those not enrolled (n = 953) continued participation in the observational cohorts. We determined risk behaviors at baseline and at 1 year, assigned a numeric score to each behavior and defined composite score as the sum of reported behaviors. We compared changes in scores over 12 months. Both observational cohorts and SiVETs saw a significant decrease in score but greatest in the SiVETs. Investigators recruiting for trials from these populations should consider the likely effect of reduction in risk behaviors on incident HIV infection and trial statistical power.

**Electronic supplementary material:**

The online version of this article (10.1007/s10461-020-02838-w) contains supplementary material, which is available to authorized users.

## Introduction

According to UNAIDS, 1.8 million new HIV infections occurred globally in 2017, 66% of which were in Sub Saharan Africa (SSA) [1]. Available HIV prevention methods have had limited effect in curbing new HIV infections in SSA because of poor adherence and/or lack of access [2]. Three possible long-term hopes for controlling the HIV pandemic are an effective and affordable HIV vaccine [3], a long-acting drug [4], and antibody injection [5]. Successful efficacy trials will need populations with high HIV incidence and SSA is likely to be a key destination for many such trials. However, many SSA countries suffer from generalized HIV epidemics [6, 7], and although the HIV incidence is below 1% per annum [8], the HIV prevalence in the general population in Uganda has consistently remained above 5% [1]. In such a setting, trials may not be conducted in the general population but population sub groups.

Occupational subpopulations, such as Fisherfolks (FF) and female sex workers (FSW), are suitable for HIV vaccine efficacy trials [9–12]. The incidence of HIV is much higher in these subpopulations, with incidence rates as high as 11 per 100 persons at risk in Uganda [9–14]. These groups have shown high willingness to participate in HIV prevention research [15, 16] and have good retention in study follow up [17, 18]. However, most incidence and retention information comes from observational cohorts, and trials often have lower HIV incidence than observational cohorts drawn from the same population [9, 19, 20]. In 2007/8, lower than expected HIV incidence led to the premature termination of three microbicides trials in West Africa [20–22].

Two key reasons have been put forward to explain the reduced HIV incidence in trials. First, an inclination for participants to reduce risky behaviors due to vigorous trial HIV risk-reduction measures. Second, there may be important differences between participants who join clinical trials and those that do not [20–22]. In such trials, participants have reported increased condom use, fewer sexual partners, and fewer sex acts compared to their baseline behavior.

To our knowledge, no HIV efficacy trials to date have completed follow up among FF on the shoreline of Lake Victoria nor among FSW in Kampala. Observational studies in FF and FSW in Uganda have shown very high HIV risk behaviors and genital infections [12, 17, 23–25]. HIV incidence in these groups has also been high [12, 14]. As an ethical requirement, conduct of HIV vaccine efficacy trials requires that participants receive HIV behavioral risk reduction messages/measures and this is likely to decrease the proportion of participants who engage in high-risk behavior.

Composite sets of HIV risk components have been previously used in cohorts of serodiscordant couples in seven African countries [26] and Men who have sex with men in China [27], Kenya [28] and Brazil [29], to generate HIV risk scores. In these studies, a lower risk score was associated with 20 to 85% [26, 29] lower HIV incidence. The composite score allowed for more precise predictive capability of risk on HIV incidence, than individual predictors [26].

Since 2008, the International AIDS Vaccine Initiative (IAVI) in collaboration with MRC/UVRI and LSHTM Uganda research Unit have run cohorts of FF and FSW [10, 11, 17, 18, 24]. Beginning July 2012, HIV simulated vaccine efficacy trials (SiVETs) (designed to mimic an HIV vaccine efficacy trial using a commercially licensed Hepatitis B vaccine) were nested within both cohorts [9, 13]. Results from these studies have shown a 50% reduction in HIV incidence in the simulation trials compared to the cohorts in which they were nested, despite the fact that the licensed vaccine has no effect on HIV infection [9, 13].

We use data from the two observational cohorts and the nested SiVETs to: (i) determine the proportion of participants with decreased composite risk score at end of follow up, (ii) compare the decrease in composite risk score between the SiVET and the observational cohorts and (iii) determine baseline factors associated with decrease in composite risk score.

## Methods

### Study Design

Data presented in this paper come from two observational cohorts, OBC_1_ (Jan 2012–Apr 2015) in FF and OBC_2_ (Apr 2008–Apr 2017) in FSW, and two HIV simulated vaccine efficacy trials, SiVET_1_ (Jul 2012–Apr 2014) nested in OBC_1_ and SiVET_2_ (Aug 2014–Apr 2017) nested in OBC_2_.

### Description of Cohorts

#### Observational Cohorts Before SiVETs

Eligible Fisherfolks (HIV negative, aged 18–49 years, at high risk of HIV infection) were enrolled into OBC_1_ at a clinic located in Masaka town (100 km Southwest of Kampala, the capital of Uganda) about 50 km inland from the fishing communities on Lake Victoria. High risk was defined as any one of: multiple or casual sexual partners; presence of a sexually transmitted infection; non-condom use with causal partner; and alcohol use). Enrolled participants were primarily scheduled for quarterly HIV counselling and testing (HCT) and six-monthly HIV behavioral risk assessment. OBC_2_ enrolled eligible female sex workers (HIV negative, aged 18–49 years) at a clinic located in Kampala city about 2 km from the city center. The follow up schedules and reason (HIV incidence and creating a pool of participants to enroll in future HIV prevention trials) for establishing this cohort were similar to those of OBC_1_, except that HIV behavioral risk assessment in this cohort was done annually. Details of both cohorts have been previously reported [11, 13, 17, 24, 30].

#### SiVET Cohorts

**Fig. 1:**
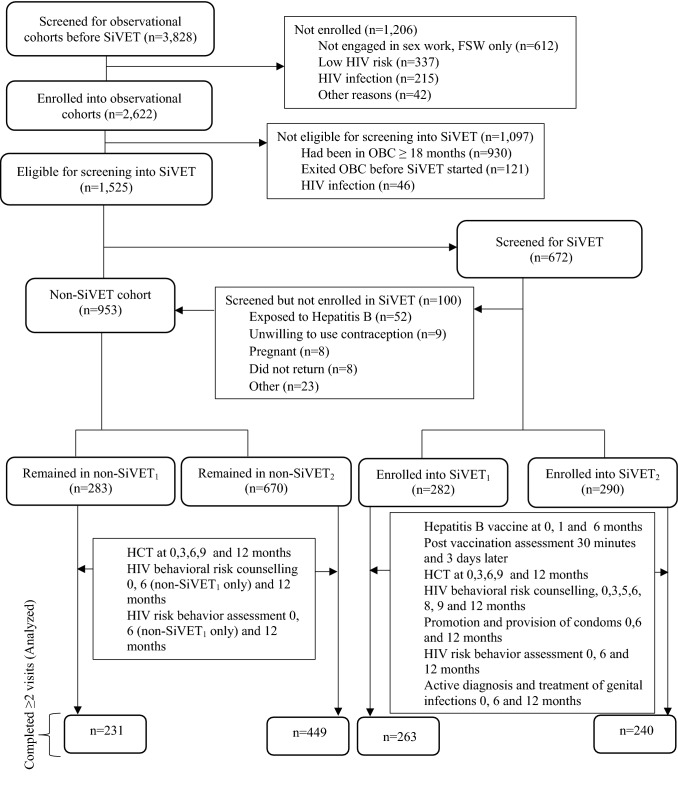
Study profile for participants screened and enrolled in SiVET cohorts and those remaining in the non-SiVET cohorts in the FF and FSW populations, Uganda

From July 2012, participants that had spent between 3 and 18 months in follow up in OBC_1_ were screened for eligibility (Table 1) and enrolled into SiVET_1_. In addition to the procedures in OBC_1_, participants in SiVET_1_ were administered a commercially licensed hepatitis B vaccine (ENGERIX-BTM GlaxoSmithKline Biologicals Rixensart, Belgium) following the standard schedule of 0, 1 and 6 months mimicking an actual HIV vaccine efficacy trial with extra follow up visits (Fig. 1). Similar procedures were followed to establish SiVET_2_, nested within OBC_2_. In both SiVETs, data were collected on risk factors, including sexual behaviors at enrolment, 6 and 12 months. The primary purpose of SiVET was to determine study participants’ retention at 12 months of follow up in a trial environment. Details of both SiVETs have been previously reported [9, 13, 30].

**Table 1:** Screening and enrolment eligibility criteria for SiVETs and non-SiVETs cohorts

SiVET cohort	Non-SiVET cohort
*Inclusion* At least 3 and no more than 18 months of follow up in the OBC_1_ or OBC_2_ HIV-1 negative and willing to undergo HIV testing Age 18 to 49 years Able and willing to provide written informed consent Able and willing to provide adequate locator information including physical address Willing and able to return for follow-up clinic visits Intending to reside in study area for at least 1 year Willing to undergo pregnancy testing Not breastfeeding and no intent for pregnancy in the next year Willing to use effective contraception during the study and at least 3 months after the last vaccination	*Inclusion* At least 3 months and no more than 18 months of follow up in OBC_1_ or OBC_2_ Still in active follow up in the OBCs HIV-1 negative and willing to undergo HIV testing
*Exclusion* History of severe allergic reaction to any substance An acute or chronic illness Contraindication for Hepatitis B vaccine Participation in another clinical trial Hepatitis B positive (only SiVET_2_)	*Exclusion* HIV positive

*SiVET* simulated vaccine efficacy trial, *OBC* observational cohort

#### Non-SiVET Cohorts (Observational Cohorts in the SiVET Concurrent Period)

Non-SiVET_1_ in FF and non-SiVET_2_ in FSW cohorts comprised of participants in the respective OBCs that either failed the SiVET screening procedure (Table 1 and Fig. 1) or who were not enrolled because the SiVET had reached its target sample size. In both non-SiVET cohorts, data were collected on sexual behaviors at enrolment, 6 months (only non-SiVET_1_) and 12 months.

#### HIV Risk Components Score

We defined a composite risk score for each participant taking account of the following: alcohol consumption; use of alcohol prior to sex; number of sexual partners; starting a new sexual relationship recently; condom use; and presence of genital discharge and/or disease, with scoring as shown in Table 2. A higher score indicates higher risk components. We used the difference in this composite score between baseline and end of follow up (12 months) as a measure of change in risk components [29], where a positive value indicates an increase in high-risk behavior.

**Table 2:** HIV risk reduction measures and risk score determination in the non-SiVET and SiVET cohorts in the key populations, Uganda

Risk reduction measure	Study cohort		Assessment question	Component score
	SiVET	non-SiVET		
HIV counselling and testing	Yes	Yes	HIV test results^a^	na
Counselling on alcohol consumption	Yes	No	Alcohol consumption (last 3 months)^a^	Never (0) Sometimes (1) Weekly (2) Daily (3)
Counselling on having sex under influence of alcohol	Yes	No	Having sex under influence of alcohol (last 3 months)^a^	Never (0) Sometimes (1) Frequently (2) Always (3)
Counselling on the number of sexual partners	Yes	Yes	Number of sexual partners (last 3 months)^a^	None (0) One (1) Two (2) Three (3) ≥ Four (4)
Counselling on having new (casual) sexual partners	Yes	Yes	Number of new sexual partner besides the regular (last 3 months)^a^	None (0) One (1) Two (2) Three (3) ≥ Four (4)
Promotion and provision of condoms	Yes	No (provided on request)	Condom use with a new sexual partner (last 3 months)^a^	No new partner (0) Always (1) Frequently (2) Sometimes (3) Never (4)
Active diagnosis and treatment for genital discharge (GD)	Yes	Symptomatic treatment	Presence of genital discharge^a^	No (0) Yes (1)
Active diagnosis and treatment for genital ulcer disease (GUD)	Yes	Symptomatic treatment	Presence of genital ulcer/sores^a^	No (0) Yes (1)
Total least score = 0 while the maximum worst score = 20				

*na* not applicable, *SiVET* simulated vaccine efficacy trial

^a^Schedule indicated in Fig. 1

#### Data Management and Statistical Methods

The data from non-SiVET cohorts were entered and managed in MS Access 2003 (Microsoft Corporation, Redmond, WA), and from SiVET cohorts in OpenClinica 3.5 (Waltham, MA). All data were analyzed in Stata 14.0 (Stata Corp, College Station, TX, USA). We excluded from analysis participants who did not return for at least one HIV risk assessment follow-up visit. We summarized baseline characteristics using frequencies and percentages and compared them between non-SiVET and SiVET cohorts in the same population with chi-square tests. Bar graphs were used to display (i) the proportion of participants reporting each risk component at baseline and at 12-month follow up and (ii) the proportion of participants for each reported risk component who experienced a decrease in their risk score from that reported at baseline. We categorized the score difference into a binary variable, 1 for decreased risk component (difference < 0) and 0 otherwise (difference ≥ 0). The proportion of participants with decreased risk component was estimated as the number with difference < 0 divided by the total number of participants in the analysis expressed as a percentage. We estimated the mean and median of the composite risk scores at baseline and at 12 months stratified by non-SiVET and SiVET cohort as well as the study population. We fitted linear regression models stratified by the study population to determine the relationship of risk score at 12 months with study (non-SiVET vs SiVET) or other baseline characteristics adjusted for baseline risk score. After bivariable analyses, a multivariable model was fitted. In the multivariable model, factors were removed from the model using a backward elimination algorithm retaining any factors which remained significant predictor of dropping risk score (p ≤ 0.05) or which caused a change in the regression coefficient of 20% or more (i.e., suggesting they were a confounding factor). Sex, age group and study cohort (SiVET and non-SiVET) were included a priori. We preferred linear models to Poisson or negative binomial because the data under consideration did not have any zero or skewed scores. However, we further fitted Poisson models in a supplementary analysis and similar results were observed, Supplementary Table 6.

Two sensitivity analyses were performed: one, stratifying the fisherfolk population by gender; the other comparing the primary outcome between non-SiVET participants (those not screened because of SiVET recruitment accrual) to (a) SiVET screen failures and (b) SiVET.

## Results

### Screening, Enrolment and Follow Up

In total, 3828 volunteers were screened and 2622 (68%) enrolled into observational cohorts before SiVETs, Fig. 1. At the start of the SiVET period, 1525 (58%) of those enrolled into the original observational cohorts were eligible for screening into SiVETs, 672 (44%) were consecutively screened until 572 (85%) were enrolled. This analysis includes data from the 1183 participants who completed at least one follow-up behavior assessment visit: 231 (81.6%) of the participants in the non-SiVET_1_ cohort, 449 (65.1%) non-SiVET_2_, 263 (93.3%) SiVET_1_ and 240 (82.8%) SiVET_2_ (Fig. 1).

### Baseline Characteristics of the Analyzed Participants

FF population: From the counts and percentages, compared to the non-SiVET_1_ cohort, the SiVET_1_ cohort had more men (73% vs 50%), more participants aged 35+ years (25% vs 14%), more participants engaged in fishing or related occupations (59% vs 45%) and more participants who had lived at their current location for more than 1 year (83% vs 70%).

FSW population: From the counts and percentages, compared to the non-SiVET_2_ cohort, the SiVET_2_ cohort had more participants aged 35+ years (24% vs 14%), more with secondary or higher education (44% vs 17%), and more participants who had lived at the current location for one or more years (85% vs 65%). See Table 3 for more details.

**Table 3:** Baseline characteristics of participants in the non-SiVET and SiVET cohorts in the key populations in Uganda, counts, percentages and chi-squared test

Variable	Total (%)	FF (N = 494)			FSW (N = 689)		
		Non-SiVET1 n (%)	SiVET1 n (%)	p-value	Non-SiVET2 n (%)	SiVET2 n (%)	p-value
Overall	1183 (100)	231 (100)	263 (100)		449 (100)	240 (100)	
Sex				< 0.01			
Male	306 (26)	115 (50)	191 (73)		–	–	
Female	877 (74)	116 (50)	72 (27)		449 (100)	240 (100)	
Age (years)				0.01			< 0.01
18–24	440 (37)	104 (45)	79 (30)		191 (43)	66 (28)	
25–34	522 (44)	94 (41)	119 (45)		193 (43)	116 (48)	
35+	221 (19)	33 (14)	65 (25)		65 (14)	58 (24)	
Ethnicity				0.02			0.07
Baganda	544 (46)	94 (41)	121 (46)		204 (45)	125 (52)	
Banyankole	170 (14)	40 (17)	27 (10)		76 (17)	27 (11)	
Banyarwanda	150 (13)	59 (26)	53 (20)		21 (5)	17 (7)	
Other	319 (27)	38 (16)	62 (24)		148 (33)	71 (30)	
Religion				0.36			0.98
Christian	899 (76)	172 (74)	205 (78)		340 (76)	182 (76)	
Muslim	284 (24)	59 (26)	58 (22)		109 (24)	58 (24)	
Education				0.12			< 0.01
None	237 (20)	25 (11)	17 (6)		182 (41)	13 (5)	
Primary	666 (56)	156 (67)	197 (75)		191 (42)	122 (51)	
Secondary+	280 (24)	50 (22)	49 (19)		76 (17)	105 (44)	
Marital status				0.24			0.01
Single never married	359 (31)	67 (29)	75 (29)		158 (35)	59 (25)	
Married	275 (23)	104 (45)	135 (51)		24 (5)	12 (5)	
Single ever married	549 (46)	60 (26)	53 (20)		267 (60)	169 (70)	
Occupation				< 0.01			0.22
Small scale business	147 (12)	54 (23)	70 (27)		13 (3)	10 (4)	
Fishing/related	259 (22)	104 (45)	155 (59)		–	–	
Hotel/bar/hair saloon	298 (25)	41 (18)	22 (8)		144 (32)	91 (38)	
Sex work	425 (36)	–	–		289 (64)	136 (57)	
Other	54 (5)	32 (14)	16 (6)		3 (1)	3 (1)	
Duration (years) in community				< 0.01			< 0.01
0–1	306 (26)	70 (30)	44 (17)		156 (35)	36 (15)	
> 1	877 (74)	161 (70)	219 (83)		293 (65)	204 (85)	
Illicit drug use				0.53			0.95
No	572 (48)	207 (90)	231 (88)		87 (19)	47 (20)	
Yes	611 (52)	24 (10)	32 (12)		362 (81)	193 (80)	

*FF* Fisherfolk, *FSW* female sex worker, *SiVET* simulated vaccine efficacy trial

### Risk Indicator Characteristics at Baseline and 12 Months

**Fig. 2:**
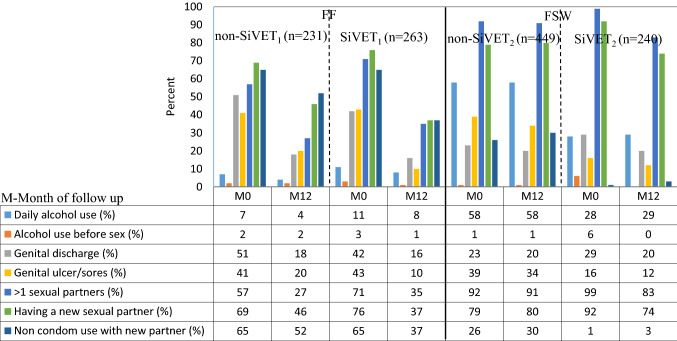
Proportion of risk component measures at baseline and 12 months in the non-SiVET and SiVET cohorts among the key populations in Uganda

Reported participant behavior/characteristics at baseline and 12 months are shown in the bar graph, Fig. 2. For the FF population, the baseline components were broadly comparable between the non-SiVET_1_ and SiVET_1_ cohorts, except for the proportion of participants reporting more than one sexual partner, which was higher in the SiVET_1_ (71%) compared to the non-SiVET_1_ (57%). At 12 months of follow up, the two groups were largely similar, except for having genital ulcer/sores (20% vs 10%), reporting new sexual partners (46% vs 37%) and non-condom use with new sexual partner (52% vs 37%) that were all higher in non-SiVET_1_ compared to SiVET_1_. Similarly, in the FSW population the baseline components were comparable between the non-SiVET_2_ and SiVET_2_ populations, except for reported daily alcohol use (58% vs 28%), genital ulcer/sores (39% vs 16%) and non-condom use with new sexual partner (26% vs 1%) that were all higher in the non-SiVET_2_ (Fig. 2). At 12 months of follow up, the differences between non-SiVET_2_ and SiVET_2_ seen at baseline remained.

### Composite Risk Score

The composite risk scores for each cohort, and stratified by study population, are shown by means and medians in Table 4. In both cohorts, the mean risk score was higher in the SiVET than the corresponding non-SiVET at baseline; in the FF population, this situation had reversed in the 12 months of follow up.

**Table 4:** Risk score at baseline and 12 months of follow up stratified by study cohort and population (means and medians)

Population	Study	Risk score at baseline				Risk score at 12 months			
		Mean	SD	Median	IQR	Mean	SD	Median	IQR
FF	Non-SiVET_1_	7.7	3.9	8	5–10	5.1	3.6	4	2–7
	SiVET_1_	8.8	3.6	9	6–11	4.8	3.2	5	2–7
FSW	Non-SiVET_2_	8.7	2.7	9	7–10	8.5	2.5	9	7–10
	SiVET_2_	11.4	3.1	9	8–13	9.5	3.8	10	7–12

*FF* Fisherfolk, *FSW* female sex worker, *SiVET* simulated vaccine efficacy trial, *SD* standard deviation, *IQR* interquartile range

### Decrease in Risk Score Between Baseline and 12 Months of Follow-Up

Overall, 170 (73.6%) of the participants in the non-SiVET_1_ and 214 (81.4%) in the SiVET_1_ cohort in the FF population experienced a decrease in risk score (p = 0.038). Similarly, 197 (43.9%) of the participants in the non-SiVET_2_ compared to 149 (62.1%) in SiVET_2_ cohort in the FSW population experienced a decrease in risk score, p < 0.001.

**Fig. 3:**
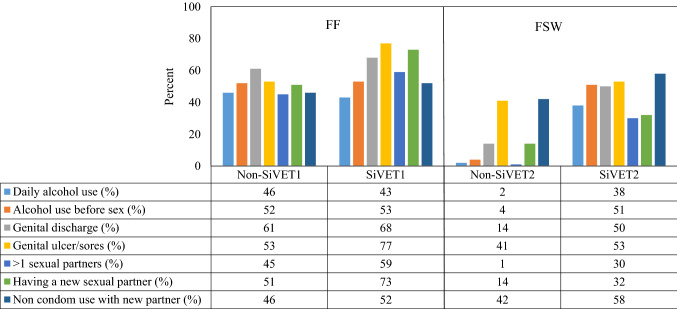
Proportion of participants with decrease in the score of a given risk component measure between baseline and 12 months among the key populations in Uganda

The bar graph, Fig. 3 shows the proportion of participants whose individual component risk scores at 12 months decreased from that at baseline. In the FF population, there was generally a large decrease, of 40% or more, in the risk score for all components in both non-SiVET_1_ and SiVET_1_. The difference between non-SiVET_1_ and SiVET_1_ cohorts were observed mainly in the proportion with decreased genital ulcer/sores (53% vs 77%) and those reporting new sexual partners (51% vs 73%).

In the FSW population there were generally smaller decreases (typically less than 15%) in the risk score in the non-SiVET_2_ for most components except for genital ulcer/sores and non-condom use with a new sexual partner that declined by about 40%. On the other hand, the decreases in risk score were over 30% for all components in the SiVET_2_ cohort. Comparing non-SiVET_2_ to SiVET_2_, the proportion of decreased risk score were higher in SiVET_2_ for all components.

### Regression Analysis of Risk Score at 12 Month

Table 5 shows the results of linear regression models comparing non-SiVET to corresponding SiVET cohort at 12 months of follow up adjusted for baseline risk score and other factors shown in the table. Overall, in the FF population, the predicted mean risk score for SiVET_1_ at 12 months was 0.63 points lower (95% CI – 1.18 to – 0.08, p = 0.024) than for non-SiVET_1_ after adjustment for factors shown in Table 5. In FSW it was 0.10 points lower (95% CI – 0.58 to 0.39, p = 0.692) for SiVET_2_ than non-SiVET_2_ after adjusting for factors shown in Table 5. In the FF population, the predicted mean risk score for females was 1.65 points lower (95% CI – 2.24 to – 1.05, p < 0.001) than males.

**Table 5:** Unadjusted and adjusted factors associated with decrease in risk score among key populations in Uganda, linear regression models results

Variable	FF (N = 494)				FSW (N = 689)			
	Uncoef (95%CI)	p-value	aCoef (95%CI)	p-value	Uncoef (95%CI)	p-value	aCoef (95%CI)	p-value
Study								
Non-SiVET	Ref		Ref		Ref		Ref	
SiVET	– 0.63 (– 1.18 to – 0.08)	0.024	– 0.92 (– 1.47 to – 0.37)	0.001	– 0.10 (– 0.58 to 0.39)	0.692	– 0.12 (0.63 to 0.38)	0.625
Sex								
Male	Ref		Ref		–	–	–	–
Female	– 1.55 (– 2.11 to – 0.98)	< 0.001	– 1.65 (– 2.24 to – 1.05)	< 0.001				
Age (years)								
18–24	Ref		Ref		Ref		Ref	
25–34	0.25 (– 0.36 to 0.86)	0.425	0.09 (– 0.51 to 0.69)	0.764	0.09 (– 0.38 to 0.56)	0.697	0.09 (– 0.41 to 0.59)	0.729
35 +	– 0.23 (– 0.99 to 0.52)	0.544	– 0.24 (– 0.99 to 0.50)	0.522	0.07 (– 0.54 to 0.69)	0.810	0.14 (– 0.52 to 0.79)	0.686
Ethnicity								
Baganda	Ref		Ref		Ref			
Banyankole	0.65 (– 0.20 to 1.50)	0.132	0.43 (– 0.40 to 1.25)	0.310	– 0.26 (– 0.89 to 0.36)	0.412		
Banyarwanda	– 0.26 (– 0.96 to 0.45)	0.478	– 0.14 (– 0.83 to 0.55)	0.694	– 0.09 (– 1.04 to 0.86)	0.849		
Other	0.39 (1.13 to 2.55)	0.300	0.22 (– 0.50 to 0.93)	0.553	0.35 (– 0.14 to 0.83)	0.159		
Religion								
Christian	Ref				Ref			
Muslim	– 0.31 (– 0.96 to 0.33)	0.337			– 0.01 (– 0.51 to 0.49)	0.968		
Education								
None	Ref				Ref			
Primary	– 0.10 (– 1.09 to 0.89)	0.842			0.26 (– 0.25 to 0.77)	0.317		
Secondary +	– 0.55 (– 1.67 to 0.57)	0.337			– 0.15 (– 0.72 to 0.43)	0.614		
Marital status								
Single never married	Ref				Ref		Ref	
Married	– 0.42 (– 1.07 to 0.22)	0.197			– 1.03 (– 2.03 to – 0.04)	0.042	– 1.15 (– 2.17 to – 0.14)	0.026
Single ever married	– 0.29 (– 1.05 to 0.47)	0.456			0.003 (– 0.46 to 0.46)	0.989	– 0.14 (– 0.65 to 0.37)	0.599
Occupation								
Small scale business	Ref				Ref		Ref	
Fishing/related	0.64 (– 0.03 to 1.31)	0.060			–		–	
Hotel/bar/salon	– 0.37 (– 1.30 to 0.56)	0.434			– 0.59 (– 1.80 to 0.62)	0.339	– 0.56 (– 1.78 to 0.66)	0.368
Sex work					– 0.21 (– 1.40 to 0.97)	0.726	– 0.16 (– 1.35 to 1.04)	0.798
Other	– 0.48 (– 1.50 to 0.55)	0.360			– 1.33 (– 3.87 to 1.21)	0.304	– 1.27 (– 3.82 to 1.27)	0.326
Duration (years) in community								
0–1	Ref				Ref		Ref	
> 1	0.41 (– 0.24 to 1.05)	0.217			0.30 (– 0.17 to 0.77)	0.213	0.31 (– 0.18 to 0.80)	0.209
Illicit drug use								
No	Ref		Ref		Ref			
Yes	1.06 (0.19–1.94)	0.017	0.78 (– 0.07 to 1.63)	0.073	– 0.29 (– 0.82 to 0.24)	0.288		

*FF* Fisherfolk, *FSW* female sex worker, *SiVET* simulated vaccine efficacy trial, *CI* confidence interval, *Uncoef* unadjusted linear regression model coefficient, *aCoef* adjusted linear regression model coefficient, *p value* statistical significance, *Ref* reference category

### Results of the sensitivity analyses

Linear regression models comparing non-SiVET participants (not screened because of SiVET recruitment accrual) to SiVET and SiVET screen failures and adjusting for the factors in Table 5 were applied separately to each of the two sub-populations. Compared to the non-SiVET participants in the FF population; the predicted mean risk score was 0.75 points lower (95% CI – 1.31 to – 0.20, p = 0.004) in SiVET participants, and 1.94 lower (95% CI – 3.60 to – 0.29, p = 0.021) in SiVET screen failures. Similarly, in the FSW compared to non-SiVET participants, the predicted mean risk score was 0.05 points lower (95% CI – 0.57 to 0.46, p = 0.836) in SiVET participants but 0.52 points higher (95% CI – 0.27 to 1.32, p = 0.198) in the SiVET screen failures.

In a further sensitivity analysis of the adjusted linear regression models stratified by sex in the FF population, comparing non-SiVET participants to SiVET ones, the predicted mean risk score for SiVET was 1.24 points lower (95% CI – 2.01 to – 0.48, p = 0.002) for the men and 0.67 points lower (95% CI – 1.41 to – 0.08, p = 0.080) for the women. All results and adjustment risk factors are shown in Supplementary Table 7.

## Discussion

In this paper, we compared behaviors of people recruited into simulated HIV vaccine efficacy trials with people who remained in the observational cohorts in which the trials were nested. The cohorts consisted of fisherfolks and female sex workers in Uganda. We found that the proportion of participants whose composite HIV risk score decreased was higher among participants who enrolled in SiVETs. Generally, the proportion of participants with decreased risk score were lower among FSW than FF; conversely, the difference between SiVET and non-SiVET cohorts was greatest in the FSW population. The results from the linear regression analysis suggested that participation in a SiVET was independently associated with a decrease in composite risk score in both populations; however, there was only good statistical evidence for this among FF. This result is consistent with previous trials, which reported participants’ engagement in lower HIV risky behaviors during trial follow up beyond that observed in the source population [20–22].

In the FF population, women were more likely than men to report a decrease in HIV risk behaviors. Literature shows that women in Sub Saharan Africa [31] have better health seeking behaviors and they could have been more likely to respond to the HIV risk reduction measures provided in these cohorts.

Although the observational cohorts were the recruitment source for the SiVETs, screening and enrollment was consecutive and not random; thus participants’ baseline characteristics between SiVET and non-SiVET cohorts differed in some important ways in both populations. SiVETs recruited more men (SiVET_1_ in FF), more participants aged 35 or over, more educated participants (SiVET_2_ in FSW) and more people who had lived in the community for longer than 1 year. Previous studies have highlighted the significant selection differences between clinical trials and source population and its effect on the trial outcomes [19–21, 32].

Clinical trials of active interventions have shown a 50% to 78% reduction in HIV incidence in the control arm compared to that predicted from the source population [20–22]. This led to many of these trials ending early due to futility. Similarly, previous publications from these SiVETs [9, 13] in FF and FSW populations have indicated a 40% to 50% reduction in HIV incidence in those recruited into the trial compared to the source population, even though the Hepatitis B vaccine used in the SiVETs had no effect on HIV susceptibility.

It is possible that consecutive screening and enrolment into SiVET included more of the participants that were likely to report on time for study visits and adhere to HIV risk reduction measures. The engagement with less risky behaviors might lower the risk for HIV infection in intervention trials for reasons unrelated to the product being tested. In the FF population, individual HIV risk components generally decreased between baseline and 12 months, more so in the SiVET cohorts. More notable was a decrease in ‘condomless’ sex with a new sexual partner. This was more marked in the SiVET, about 43% decrease as opposed to 20% in non-SiVET cohort. Though not documented at interim clinic visits, SiVET participants had more access to condoms because of the more clinic visits.

On the other hand, in the FSW population, there were marginal decreases in individual reported risk behavior in the SiVET cohort and very minimal to none in the non-SiVET cohort. This could be associated with the occupational demands of sex work as the livelihood of 100% of these cohort participants depended on high-risk behavior. Unlike the FF population, the FSW population was comprised of females and only male condoms were provided for use with male sexual clients. Literatures in Africa shows that, females have limited power in relationships to demand condom use [33]. Furthermore, studies in female sex workers population in Africa [34] and elsewhere [35–37] have shown that ‘condomless’ sex attracted more pay. This could hamper decreases in ‘condomless’ sex with new or other causal sexual partners as seen in this population.

Our analysis has a number of strengths that included a reasonable sample size, two distinct key populations in which SiVET and non-SiVET cohorts were aligned to a set duration of time. Both SiVET and corresponding non-SiVET cohorts’ participants were seen at the same clinic by the same study staff under standardized study procedures. All staff were trained on both studies, and study visits and conduct were done per Standard Operating Procedures to assure data were collected in a systematic manner. Our comparative analysis is not without limitations, however. SiVET cohorts were more likely to screen and enroll participants that reported on time for their 3 to 18 months source cohort clinic visit. It is possible that timely participants are also more inclined to take up the HIV behavioral risk reduction measures or are otherwise more compliant with study instructions. The study procedures in the SiVET and non-SiVET cohorts were not blinded. However, at the time of the conduct of SiVETs, the primary aim was not to compare SiVET to non-SiVET participants and if there were any differences in the conduct of study procedures, they were likely modest unconscious biases, and are unlikely to have affected the outcomes considered in this analysis. Participants were encouraged to take more condoms in case their stock was finished before the next scheduled clinic visits and we did not document the data on condom demands on visits that HIV risk behavior assessment was not scheduled. This could have helped explain the more marked increase in condom use with a new sexual partner seen in the SiVETs cohort because participants in this cohort had more of such visits. Notwithstanding these limitations, our comparative analysis gives a rare opportunity of estimating the likely drop in HIV risk components in trials nested within source cohorts in two distinct key populations.

In conclusion, results from both key populations suggest that participation in both studies positively affected risk-taking behavior, and in some cases, this was more pronounced in a “Simulation trial” conducted alongside an observational study aligned to the same duration of time. Previous publications from these populations have shown lower HIV incidence in SiVET cohorts compared to non-SiVET cohorts even when aligned to the same duration of follow up. Other studies have also shown lower HIV incidence in the trial control arm compared to that predicted from observational data at the trial on set. Therefore, it is likely that participants who join trials are mostly those likely to respond to HIV risk reduction measures beyond what is seen in source population or the general population. While the more than half drop in the HIV risk score in FF and one third in FSW participating in SiVETs is of great public health importance, investigator-recruiting participants into clinical trials from observational cohorts in these key populations need to consider the likely effect of reduction in HIV risk components on likelihood of seroconversion and the trial statistical power. Taking the results of this analysis and previous publications on HIV incidence from these SiVETs and non-SiVET cohorts, it is encouraging that these key populations could still be suitable for HIV vaccine efficacy and other HIV prevention trials.

## Electronic supplementary material

Below is the link to the electronic supplementary material.

Electronic supplementary material 1 (DOCX 21 kb)

## Data Availability

The MRC/UVRI and LSHTM Uganda Research Unit encourages open data access and has a data sharing policy accessible at https://www.mrcuganda.org/publications/data-sharing-policy. The policy summarizes the conditions under which data collected by the Unit can be made available to other bona fide researchers, the way in which such researchers can apply to have access to the data and how data will be made available if an application for data sharing is approved. Should any other researchers need to have access to the data from which this manuscript was generated, the processes to access the data are well laid out in the policy. The corresponding and other co-author emails have been provided and they could be contacted anytime for further clarifications and/or support to access the data.

## References

[1] UNAIDS. State of the epidemic. 2018.

[2] Mills EJ, Nachega JB, Buchan I, Orbinski J, Attaran A, Singh S (2006). Adherence to antiretroviral therapy in sub-Saharan Africa and North America: a meta-analysis. JAMA.

[3] Esparza J (2001). An HIV vaccine: how and when?. Bull World Health Organ.

[4] Barnhart M (2017). Long-acting HIV treatment and prevention: closer to the threshold.

[5] Margolis DM, Koup RA, Ferrari G (2017). HIV antibodies for treatment of HIV infection. Immunol Rev.

[6] AVERT. HIV and AIDS in East and Southern Africa regional overview February 2019 https://www.avert.org/professionals/hiv-around-world/sub-saharan-africa/overview. Accessed on 17 April 2019

[7] Kharsany AB, Karim QA (2016). HIV infection and AIDS in sub-Saharan Africa: current status, challenges and opportunities. Open AIDS J.

[8] Ruzagira E, Wandiembe S, Abaasa A, Levin J, Bwanika A, Bahemuka U (2011). Prevalence and incidence of HIV in a rural community-based HIV vaccine preparedness cohort in Masaka, Uganda. PLoS ONE.

[9] Abaasa A, Nash S, Mayanja Y, Price M, Fast PE, Kamali A (2019). Simulated vaccine efficacy trials to estimate HIV incidence for actual vaccine clinical trials in key populations in Uganda. Vaccine.

[10] Kamali A, Nsubuga R, Ruzagira E, Bahemuka U, Asiki G, Price M (2016). Heterogeneity of HIV incidence: a comparative analysis between fishing communities and in a neighbouring rural general population, Uganda, and implications for HIV control. Sex Transm Infect.

[11] Kasamba I, Nash S, Seeley J, Weiss HA. HIV incidence among women at high risk of HIV infection attending a dedicated clinic in Kampala, Uganda: 2008–2017. Sexually transmitted diseases. 2019. 10.1097/OLQ.0000000000000978 31095103

[12] Seeley J, Nakiyingi-Miiro J, Kamali A, Mpendo J, Asiki G, Abaasa A (2012). High HIV incidence and socio-behavioral risk patterns in fishing communities on the shores of Lake Victoria Uganda. Sex Trans Dis.

[13] Abaasa A, Asiki G, Price MA, Ruzagira E, Kibengo F, Bahemuka U (2016). Comparison of HIV incidence estimated in clinical trial and observational cohort settings in a high risk fishing population in Uganda: implications for sample size estimates. Vaccine.

[14] Kiwanuka N, Ssetaala A, Nalutaaya A, Mpendo J, Wambuzi M, Nanvubya A (2014). High incidence of HIV-1 infection in a general population of fishing communities around Lake Victoria, Uganda. PLoS ONE.

[15] Asiki G, Abaasa A, Ruzagira E, Kibengo F, Bahemuka U, Mulondo J (2013). Willingness to participate in HIV vaccine efficacy trials among high risk men and women from fishing communities along Lake Victoria in Uganda. Vaccine.

[16] Kiwanuka N, Ssetaala A, Mpendo J, Wambuzi M, Nanvubya A, Sigirenda S (2013). High HIV-1 prevalence, risk behaviours, and willingness to participate in HIV vaccine trials in fishing communities on Lake Victoria, Uganda. J Intl AIDS Soc.

[17] Bahemuka UM, Abaasa A, Ruzagira E, Lindan C, Price MA, Kamali A (2019). Retention of adults from fishing communities in an HIV vaccine preparedness study in Masaka, Uganda. PLoS ONE.

[18] Abaasa A, Asiki G, Mpendo J, Levin J, Seeley J, Nielsen L (2015). Factors associated with dropout in a long term observational cohort of fishing communities around lake Victoria, Uganda. BMC Res Notes.

[19] Pinsky P, Miller A, Kramer B, Church T, Reding D, Prorok P (2007). Evidence of a healthy volunteer effect in the prostate, lung, colorectal, and ovarian cancer screening trial. Am J Epidemiol.

[20] Feldblum PJ, Adeiga A, Bakare R, Wevill S, Lendvay A, Obadaki F (2008). SAVVY vaginal gel (C31G) for prevention of HIV infection: a randomized controlled trial in Nigeria. PLoS ONE.

[21] Peterson L, Nanda K, Opoku BK, Ampofo WK, Owusu-Amoako M, Boakye AY (2007). SAVVY®(C31G) gel for prevention of HIV infection in women: a phase 3, double-blind, randomized, placebo-controlled trial in Ghana. PLoS ONE.

[22] Peterson L, Taylor D, Roddy R, Belai G, Phillips P, Nanda K (2007). Tenofovir disoproxil fumarate for prevention of HIV infection in women: a phase 2, double-blind, randomized, placebo-controlled trial. PLoS Clin Trials.

[23] Asiki G, Mpendo J, Abaasa A, Agaba C, Nanvubya A, Nielsen L (2011). HIV and syphilis prevalence and associated risk factors among fishing communities of Lake Victoria, Uganda. Sex Transm Infect.

[24] Vandepitte J, Bukenya J, Weiss HA, Nakubulwa S, Francis SC, Hughes P (2011). HIV and other sexually transmitted infections in a cohort of women involved in high risk sexual behaviour in Kampala, Uganda. Sex Transm Dis.

[25] Hladik W, Baughman AL, Serwadda D, Tappero JW, Kwezi R, Nakato ND (2017). Burden and characteristics of HIV infection among female sex workers in Kampala, Uganda: a respondent-driven sampling survey. BMC Public Health.

[26] Kahle EM, Hughes JP, Lingappa JR, John-Stewart G, Celum C, Nakku-Joloba E (1999). An empiric risk scoring tool for identifying high-risk heterosexual HIV-1 serodiscordant couples for targeted HIV-1 prevention. J Acqu Immune Defic Syndromes.

[27] Huan X, Tang W, Babu GR, Li J, Zhang M, Liu X (2013). HIV risk-reduction counseling and testing on behavior change of MSM. PLoS ONE.

[28] Wahome E, Thiong’o AN, Mwashigadi G, Chirro O, Mohamed K, Gichuru E (2018). An empiric risk score to guide PrEP targeting among MSM in Coastal Kenya. AIDS Behav..

[29] Rocha GM, Kerr LRFS, Kendall C, Guimarães MDC (2018). Risk behavior score: a practical approach for assessing risk among men who have sex with men in Brazil. Braz J Infect Dis.

[30] Mayanja Y, Abaasa A, Namale G, Asiki G, Price MA, Kamali A (2019). Factors associated with vaccination completion and retention among HIV negative female sex workers enrolled in a simulated vaccine efficacy trial in Kampala Uganda. BMC Infect Dis.

[31] Cornell M (2013). Gender inequality: Bad for men’s health. S Afr J HIV Med..

[32] Padian NS, McLOY SI, Balkus JE, Wasserheit JN (2010). Weighing the gold in the gold standard: challenges in HIV prevention research. AIDS (London, England).

[33] Pettifor AE, Measham DM, Rees HV, Padian NS (2004). Sexual power and HIV risk, South Africa. Emerg Infect Dis.

[34] Ochako R, Okal J, Kimetu S, Askew I, Temmerman M (2018). Female sex workers experiences of using contraceptive methods: a qualitative study in Kenya. BMC Women's Health.

[35] Wang Q-q, Yang P, Gong X, Jiang J, Yang B, Yang L (2009). Syphilis prevalence and high risk behaviors among female sex workers in different settings. Chin J AIDS STDs..

[36] Yang X, Xia G (2006). Gender, work, and HIV risk: determinants of risky sexual behavior among female entertainment workers in China. AIDS Educ Prev.

[37] Yang X, Xia G, Li X, Latkin C, Celentano D (2010). Social influence and individual risk factors of HIV unsafe sex among female entertainment workers in China. AIDS Educ Prev.

