# Supplementary material for: Comparison of HIV Risk Behaviors Between Clinical Trials and Observational Cohorts in Uganda
Source: AIDS Behav. 2020 Apr 10;24(10):2872–84. doi: 10.1007/s10461-020-02838-w (PMC7467908; doi:10.1007/s10461-020-02838-w)
Supplement: Supplementary file 1 — Electronic supplementary material 1 (DOCX 21 kb) [file 10461_2020_2838_MOESM1_ESM.docx]

Supplementary Table 6: Unadjusted and adjusted factors associated with decrease in risk score in key populations in Uganda, Poisson regression models results

|  | | FF (N=494) | | |  |  | FSW (N=689) | | |  |
| --- | --- | --- | --- | --- | --- | --- | --- | --- | --- | --- |
| Variable | | uIRR (95%CI) | p-value | aIRR (95%CI) | p-value | uIRR (95%CI) | | p-value | aIRR (95%CI) | p-value |
| Study | |  |  |  |  |  | |  |  |  |
|  | Non-SiVET | Ref |  | Ref |  | Ref | |  | Ref |  |
|  | SiVET | 0.89 (0.82-0.96) | 0.003 | 0.84 (0.78-0.92) | <0.001 | 0.98 (0.93-1.04) | | 0.517 | 0.98 (0.92-1.04) | 0.465 |
| Sex | |  |  |  |  |  | |  |  |  |
|  | Male | Ref |  | Ref |  | - | | - | - | - |
|  | Female | 0.71 (0.64-0.78) | <0.001 | 0.67 (0.59-0.76) | <0.001 |  | |  |  |  |
| Age (years) | |  |  |  |  |  | |  |  |  |
|  | 18-24 | Ref |  | Ref |  | Ref | |  | Ref |  |
|  | 25-34 | 1.04 (0.95-1.14) | 0.371 | 1.01 (0.92-1.12) | 0.780 | 1.01 (0.95-1.07) | | 0.730 | 1.01 (0.95-1.07) | 0.750 |
|  | 35+ | 0.95 (0.85-1.06) | 0.371 | 0.97 (0.87-1.08) | 0.423 | 1.01 (0.94-1.08) | | 0.863 | 1.01 (0.94-1.10) | 0.738 |
| Ethnicity | |  |  |  |  |  | |  |  |  |
|  | Baganda | Ref |  | Ref |  | Ref | |  |  |  |
|  | Banyankole | 1.14 (1.02-1.28) | 0.036 | 1.11 (0.98-1.26) | 0.087 | 0.97 (0.90-1.04) | | 0.406 |  |  |
|  | Banyarwanda | 0.95 (0.85-1.05) | 0.327 | 0.97 (0.87-1.08) | 0.526 | 0.99 (0.88-1.10) | | 0.810 |  |  |
|  | Other | 1.07 (0.97-1.19) | 0.183 | 1.05 (0.94-1.17) | 0.404 | 1.04 (0.98-1.10) | | 0.197 |  |  |
| Religion | |  |  |  |  |  | |  |  |  |
|  | Christian | Ref |  |  |  | Ref | |  |  |  |
|  | Muslim | 0.94 (0.85-1.03) | 0.195 |  |  | 0.99 (0.94-1.06) | | 0.990 |  |  |
| Education | |  |  |  |  |  | |  |  |  |
|  | None | Ref |  |  |  | Ref | |  |  |  |
|  | Primary | 0.98 (0.86-1.13) | 0.831 |  |  | 1.03 (0.97-1.09) | | 0.403 |  |  |
|  | Secondary+ | 0.88 (0.75-1.04) | 0.140 |  |  | 0.98 (0.92-1.05) | | 0.606 |  |  |
| Marital status | |  |  |  |  |  | |  |  |  |
|  | Single never married | Ref |  |  |  | Ref | |  | Ref |  |
|  | Married | 0.91 (0.83-0.99) | 0.034 | 0.93 (0.84-1.03) | 0.149 | 0.88 (0.78-0.99) | | 0.047 | 0.87 (0.76-0.99) | 0.030 |
|  | Single ever married | 0.94 (0.84-1.05) | 0.263 | 1.09 (0.96-1.23) | 0.189 | 0.99 (0.95-1.06) | | 0.990 | 0.98 (0.93-1.05) | 0.601 |
| Occupation | |  |  |  |  |  | |  |  |  |
|  | Small scale business | Ref |  |  |  | Ref | |  | Ref |  |
|  | Fishing/related | 1.13 (1.02-1.25) | 0.016 | 1.03 (0.93-1.14) | 0.627 | - | | - |  |  |
|  | Hotel/Bar/Salon | 0.92 (0.80-1.07) | 0.269 | 1.06 (0.90-1.26) | 0.461 | 0.94 (0.81-1.08) | | 0.390 | 0.94 (0.81-1.09) | 0.414 |
|  | Sex work | - | - | - | - | 0.98 (0.85-1.13) | | 0.808 | 0.99 (0.86-1.14) | 0.867 |
|  | Other | 0.88 (0.74-1.04) | 0.133 | 0.96 (0.80-1.15) | 0.664 | 0.83 (0.59-1.17) | | 0.278 | 0.83 (0.59-1.17) | 0.300 |
| Duration (years) in community | | |  |  |  |  | |  |  |  |
|  | 0-1 | Ref |  |  |  | Ref | |  | Ref |  |
|  | >1 | 1.09 (0.99-1.20) | 0.092 | 1.02 (0.92-1.13) | 0.704 | 1.03 (0.98-1.09) | | 0.261 | 1.04 (0.98-1.10) | 0.231 |
| Illicit drug use | |  |  |  |  |  | |  |  |  |
|  | No | Ref |  | Ref |  | Ref | |  |  |  |
|  | Yes | 1.18 (1.05-1.32) | 0.004 | 1.12 (1.01-1.26) | 0.048 | 0.97 (0.91-1.03) | | 0.287 |  |  |

*FF-Fisherfolk, FSW-Female sex worker, SiVET-Simulated Vaccine Efficacy Trial, CI-Confidence Interval, uIRR-Unadjusted incidence rate ratio, aIRR-adjusted incidence rate ratio, p value -Statistical significance, Ref-Reference category*

Supplementary Table 7: Unadjusted and adjusted factors associated with decrease in risk score stratified by sex among fisherfolks population in Uganda, linear regression models results

|  | | Men (N=306) | | |  |  | Women (N=188) | | |  |
| --- | --- | --- | --- | --- | --- | --- | --- | --- | --- | --- |
| Variable | | unCoef (95%CI) | p-value | aCoef (95%CI) | p-value | unCoef (95%CI) | | p-value | aCoef (95%CI) | p-value |
| Study | |  |  |  |  |  | |  |  |  |
|  | Non-SiVET | Ref |  | Ref |  | Ref | |  | Ref |  |
|  | SiVET | -1.22 (-1.98 to -0.46) | 0.002 | -1.24 (-2.01 to -0.48) | 0.002 | -0.57 (-1.28 to 0.14) | | 0.114 | -0.67 (-1.41 to 0.08) | 0.080 |
| Age (years) | |  |  |  |  |  | |  |  |  |
|  | 18-24 | Ref |  | Ref |  | Ref | |  | Ref |  |
|  | 25-34 | -010 (-0.96 to 0.76) | 0.821 | -0.18 (-1.03 to 0.67) | 0.675 | 0.23 (-0.53 to 0.99) | | 0.551 | 0.38 (-0.40 to 1.16) | 0.340 |
|  | 35+ | -0.42 (-1.47 to 0.63) | 0.433 | -0.32 (-1.36 to 0.72) | 0.543 | -0.58 (-1.55 to 0.39) | | 0.240 | -0.22 (-1.22 to 0.79) | 0.675 |
| Ethnicity | |  |  |  |  |  | |  |  |  |
|  | Baganda | Ref |  | Ref |  | Ref | |  |  |  |
|  | Banyankole | 0.64 (-0.50 to 1.79) | 0.270 |  |  | 0.50 (-0.60 to 1.59) | | 0.374 |  |  |
|  | Banyarwanda | -0.21 (-1.25 to 0.84) | 0.700 |  |  | 0.18 (-0.64 to 1.01) | | 0.662 |  |  |
|  | Other | -0.07 (-1.02 to 0.88) | 0.883 |  |  | 1.00 (-0.08 to 2.08) | | 0.069 |  |  |
| Religion | |  |  |  |  |  | |  |  |  |
|  | Christian | Ref |  | Ref |  | Ref | |  |  |  |
|  | Muslim | -0.69 (-1.57 to 0.20) | 0.129 | -0.77 (-1.65 to 0.11) | 0.088 | 0.17 (-0.65 to 0.98) | | 0.685 |  |  |
| Education | |  |  |  |  |  | |  |  |  |
|  | None | Ref |  |  |  | Ref | |  |  |  |
|  | Primary | -0.44 (1.85 to 0.96) | 0.536 |  |  | -0.01 (-1.21 to 1.19) | | 0.987 |  |  |
|  | Secondary+ | -1.02 (-2.65 to 0.60) | 0.216 |  |  | -0.09 (-1.41 to 1.24) | | 0.898 |  |  |
| Marital status | |  |  |  |  |  | |  |  |  |
|  | Single never married | Ref |  |  |  | Ref | |  | Ref |  |
|  | Married | -0.45 (-1.26 to 0.37) | 0.286 |  |  | -0.25 (-1.21 to 0.70) | | 0.603 |  |  |
|  | Single ever married | 0.99 (-0.19 to 2.18) | 0.100 |  |  | -0.22 (-1.19 to 0.75) | | 0.660 |  |  |
| Occupation | |  |  |  |  |  | |  |  |  |
|  | Small scale business | Ref |  |  |  | Ref | |  | Ref |  |
|  | Fishing/related | 0.16 (-0.74 to 1.06) | 0.733 |  |  | 0.54 (-0.44 to 1.52) | | 0.278 |  |  |
|  | Hotel/Bar/Salon | -1.54 (-4.31 to 1.23) | 0.276 |  |  | 0.93 (-0.01 to 1.86) | | 0.051 |  |  |
|  | Other | -1.14 (-3.18 to 0.89) | 0.269 |  |  | 0.60 (-0.43 to 1.64) | | 0.252 |  |  |
| Duration (years) in community | | |  |  |  |  | |  |  |  |
|  | 0-1 | Ref |  |  |  | Ref | |  | Ref |  |
|  | >1 | -0.06 (-1.13 to 1.01) | 0.917 |  |  | -0.08 (-0.80 to 0.64) | | 0.836 |  |  |
| Illicit drug use | |  |  |  |  |  | |  |  |  |
|  | No | Ref |  | Ref |  | Ref | |  | Ref |  |
|  | Yes | 0.64 (-0.42 to 1.69) | 0.235 |  |  | 1.76 (0.18 to 3.34) | | 0.029 | 1.79 (0.20 to 3.39) | 0.027 |

*FF-Fisherfolk, FSW-Female sex worker, SiVET-Simulated Vaccine Efficacy Trial, CI-Confidence Interval, unCoef-Unadjusted linear regression model coefficient, aCoef-adjusted linear regression model coefficient, p value -Statistical significance, Ref-Reference category*
